# Managing recurrent rectal prolapse in women: a single-center study

**DOI:** 10.3389/fsurg.2025.1684599

**Published:** 2025-12-19

**Authors:** Stanislaw Rzadkowski, Przemyslaw Ciesielski, Malgorzata Kolodziejczak

**Affiliations:** 1 Department of General Surgery, John Paul II Western Hospital, Grodzisk Mazowiecki, Poland; 2 Department of General Surgery, Sklodowska-Curie Hospital, Ostrow Mazowiecka, Poland; 3 Warsaw Proctology Centre, St. Elizabeth’s Hospital, Warsaw, Poland

**Keywords:** rectal prolapse, Altemeier, Delorme, quality of life, ventral mesh rectopexy, resection rectopexy

## Abstract

Recurrent rectal prolapse (RRP) presents a significant clinical challenge due to the absence of standardized treatment guidelines and its impact on patients' quality of life. This retrospective study evaluates the outcomes of surgical management for RRP in a single-center cohort. We analyzed female patients (median age of 73) who underwent surgical treatment for RRP between 2014 and 2022. A total of 30 patients received either abdominal (*n* = 12) or perineal (*n* = 18) procedures, including ventral mesh rectopexy (VMR), resection rectopexy, Altemeier or Delorme techniques. Re-recurrence was more common following perineal procedures (39%) than abdominal approaches (25%). VMR and resection rectopexy demonstrated comparable success rates of 75%, while Altemeier and Delorme procedures showed 60% and 67% success, respectively. Functional outcomes improved significantly postoperatively, with the EQ-VAS quality of life score increasing from a median of 50 preoperatively to 75 at three years. Fecal incontinence and constipation scores (Wexner and CCCS) also showed marked improvement. Complications were minor and managed conservatively, with no cases of mesh erosion or anastomotic dehiscence reported. These findings support abdominal procedures, especially VMR and resection rectopexy, as the preferred treatment for medically fit patients with RRP. Perineal approaches remain viable for high-risk patients but are associated with higher recurrence. Although limited by its retrospective design, small sample size, and non-randomized treatment allocation, this study contributes valuable data to inform surgical decision-making in the management of RRP.

## Introduction

Rectal prolapse is defined as a protrusion of rectal wall through the anal canal and anus. It affects approximately 0.5% of the general population and is 6 to 9 times more common in women than in men (1). In addition to the prolapse itself, common symptoms include mucous and bloody discharge, fecal incontinence, constipation, and incomplete evacuation.

Despite its benign nature, the condition significantly impairs quality of life and often leads to social isolation and mental health issues. Surgical intervention remains the only definitive treatment.

Recurrent rectal prolapse (RRP) is a relatively frequent complication, occurring in up to 20% of cases following perineal procedures and in approximately 4%–10% after abdominal surgery (1). The recurrence rate is even higher among patients who have undergone previous surgical interventions for rectal prolapse (27% and 15% percent respectively) (2).

Although it is prevalent, there are no established guidelines to direct the optimal management of RRP. Much of the available literature is outdated, and while recent advances—particularly the development of ventral mesh rectopexy (VMR)—have broadened the range of surgical options, consensus on the best approach remains lacking. A systematic review by Houturas et al. on the surgical management of RRP (3) concluded that a treatment algorithm could not be proposed, citing the wide variation in surgical techniques and the generally low quality of evidence from different studies.

As in a primary prolapse repair, the surgical approach to RRP is either perineal or abdominal. Perineal procedures are preferred in elderly patients with frailty syndrome (4), whereas abdominal approach surgeries, which are associated with a lower recurrence rate, are the treatment of choice in younger patients (5).

The aim of this study is to present the outcomes of a single-center cohort and to contribute data that may support future strategies for the management of recurrent rectal prolapse.

## Materials and methods

### Patients

From 2014 to 2022, 32 surgically treated patients were evaluated. In the study were enrolled patients over 18 years old with recurrent full-thickness rectal prolapse (reappearance of external full-thickness prolapse).

Patients lost to follow-up (*n* = 2) were excluded from the study. Thus, eventually 30 patients were enrolled in the study—30 females and 0 men, with the median age of 73 years.

Patients with recurrent rectal prolapse included referrals from other units as well as patients who had been previously treated by the authors. Earlier treatment for rectal prolapse involved abdominal operations in 23% of patients (*n* = 7), while 70% (*n* = 21) underwent perineal procedures. Two people (7%) had undergone an indistinct procedure (Table 1).

**Table 1:** Demographics.

Previous procedure	Median age of the patient	% Female	Median BMI	Median ASA	Work (physical vs. non-physical)
Perineal (21)	78	100	25.0	2.0	48% physical
Abdominal (7)	47	100	27.5	2.0	43% physical
Indistinct (2)	64	100	26.0	1.5	50% physical

All patients included in the study were operated by one specialist team in multiple hospitals in Poland—mainly Warsaw Center for Proctology and Maria Sklodowska-Curie District Hospital in Ostrow Mazowiecka.

The analyzed group contained both patients with first recurrence, as well as more than one. The patients with recurrent rectal prolapse were *de novo* involved in the study.

The study is retrospective in nature and was approved by the bioethics committee of Regional Medical Chamber in Warsaw. Each participant was given the opportunity to decline participation in the study.

### Data collection and principles of surgical techniques

Validated questionnaires were used to evaluate functional outcomes preoperatively and again at 12 and 36 months postoperatively, with data collected during follow-up visits or through phone survey.

Patient variables analyzed included age, sex, body mass index (derived from weight and height), American Society of Anesthesiologists (ASA) physical status classification, the number and nature of prior surgical interventions for rectal prolapse. The quality of life was measured quantitively with EQ-VAS score. The presence of constipation and fecal incontinence were assessed with Cleveland Clinic Constipation Score (CCCS) and Cleveland Clinic Fecal Incontinence Severity Scoring System (Wexner score) respectively. Outcome data included operation time, 30-day mortality and re-recurrence. Post-operative complications were classified according to the Clavien-Dindo classification. Re-recurrence was defined as reappearance of external full-thickness prolapse after surgery.

Operative methods were categorized into abdominal (*n* = 12) and perineal (*n* = 18) approaches.

The preoperative protocol comprised a single prophylactic dose of intravenous antibiotics (cefazolin 1–2 g, with an additional 500 mg metronidazole administered for resectional procedures). Bowel preparation consisted of a double enema given at 4:00 p.m. and 8:00 p.m. on the day preceding surgery.

The **abdominal approach** included ventral mesh rectopexy (*n* = 8) and resection rectopexy (Frykman-Goldberg procedure) (*n* = 4). In cases of concomitant pelvic organ prolapse, sacrocolpopexy or sacrohysteropexy was performed concurrently (*n* = 2). In ventral mesh rectopexy, a B-shaped implant was fixed in a diamond-shaped configuration using 5–7 absorbable sutures (MonoPlus® 3/0) to the anterior rectal wall and with 3 non-absorbable sutures (PremiCron® 3/0) to the presacral fascia below the sacral promontory. Meshes used included polypropylene (Optilene® Mesh), bioresorbable (Phasix™ Mesh), or porcine-derived (XenMatrix™) materials. In resection rectopexy, the anastomosis was performed using a 33 mm circular stapler in a side-to-side, antiperistaltic fashion. The rectopexy itself was typically secured with 3–4 interrupted absorbable Novosyn® sutures. No mesh or protective ileostomy was employed in resection recopexy procedure.

The **perineal approach** consisted of the Delorme (*n* = 3) and Altemeier (*n* = 15) procedures. The Delorme procedure was primarily used for prolapses less than 8 cm with a high take-off, while the Altemeier procedure was preferred for prolapses exceeding 8 cm or those with a low take-off. In the Delorme procedure, eight separate absorbable sutures were used for muscle plication, and approximately 24 sutures (MonoPlus® 3/0) were placed for the mucosal anastomosis. In the Altemeier procedure, the anastomosis was constructed in a single-layer, interrupted fashion using absorbable 3/0 sutures (MonoPlus® 3/0), with an average of 24 sutures required to complete the anastomosis. In cases where a wide pelvic hiatus was present, both procedures were supplemented with posterior levatoroplasty—performed through the same incision for Altemeier, and via a separate incision for Delorme.

### Statistical analysis

Patients were stratified into two groups based on surgical approach (abdominal vs. perineal). Continuous variables were tested for normality using the Shapiro–Wilk test. Normally distributed data were expressed as mean ± standard deviation (SD) and compared using the independent-sample *t*-test. Non-normally distributed data were reported as medians with interquartile ranges (IQR) and compared using the Mann–Whitney *U* test. Categorical variables were presented as counts and percentages, with group differences assessed using Fisher's exact test or Pearson's *χ*^2^ test as appropriate. Changes in functional scores (EQ-VAS, CCCS, and Wexner) over time were assessed using paired *t*-tests or Wilcoxon signed-rank tests (Bonferroni-adjusted), depending on data distribution. A *p*-value < 0.05 was considered statistically significant. All statistical analyses were performed using R version 4.5.1 (R Core Team 2025).

## Results

### Recurrence rates

**Figure 1:**
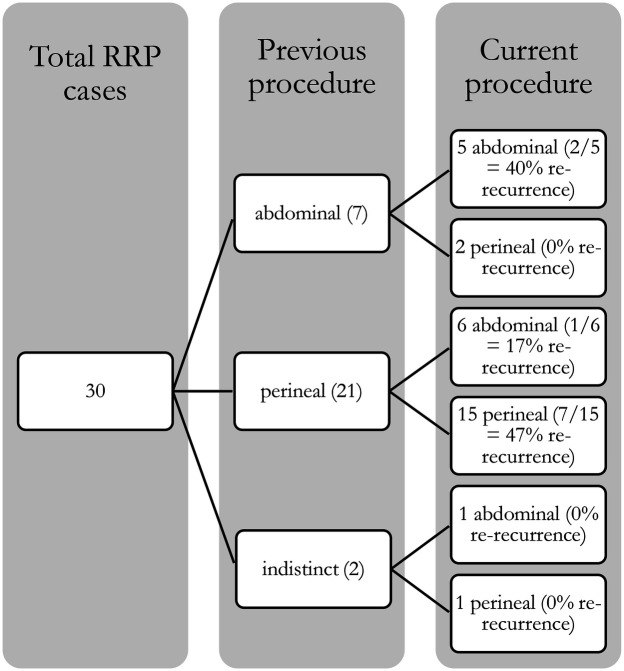
Total RRP cases.

In this study the overall re-recurrence rate at one year was 30% (9/30), with 25.0% (2/8) in the abdominal group and 32% (7/22) in the perineal group (Fisher's exact *p* = 1.00; OR = 1.38, 95% CI, 0.18–17.43). By three years, one additional re-recurrence (3.3%) was observed in the perineal group (4.5%)—too few for formal testing. Abdominal procedures demonstrated consistent success rates of 75%, with 6 out of 8 successful outcomes for ventral mesh rectopexy (VMR) and 3 out of 4 for resection rectopexy. Among perineal procedures, the Altemeier procedure had a success rate of 60% (9 out of 15), while the Delorme procedure showed a success rate of 67% (2 out of 3). The results are summarized in the Figure 1 (Total RRP cases).

### Factors associated with 1-year re-recurrence

To explore whether basic demographic or anthropometric variables differed by outcome, we first compared age and BMI between patients with and without re-recurrence at 1 year. We then entered those same variables into a multivariate logistic regression to see if either independently predicted recurrence.

There were no significant differences in age or BMI between patients with vs. without re-recurrence at 1 year (Table 2).

**Table 2:** Demographic and anthropometric comparisons.

Variable	No recurrence (mean ± SD)	Recurrence (mean ± SD)	*t*	*p*-value
Age (years)	67.24 ± 7.9	65.67 ± 8.6	0.165	0.872
BMI (kg/m^2^)	26.20 ± 3.2	25.70 ± 3.5	0.264	0.797

In a model including demographic factors (age and BMI), none of the factors significantly predicted 1-year re-recurrence (Table 3).

**Table 3:** Multivariate logistic regression.

Predictor	OR	95%CI	*p*-value
Age (per year)	0.99	0.93–1.04	0.589
BMI (per kg/m^2^)	0.93	0.71–1.20	0.592

### Functional outcomes

**Figure 2:**
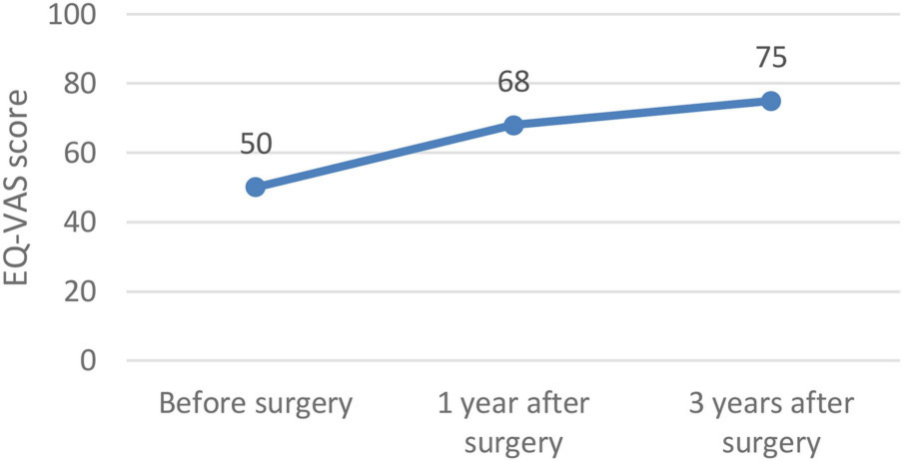
Functional outcomes – EQ-VAS score.

**Figure 3:**
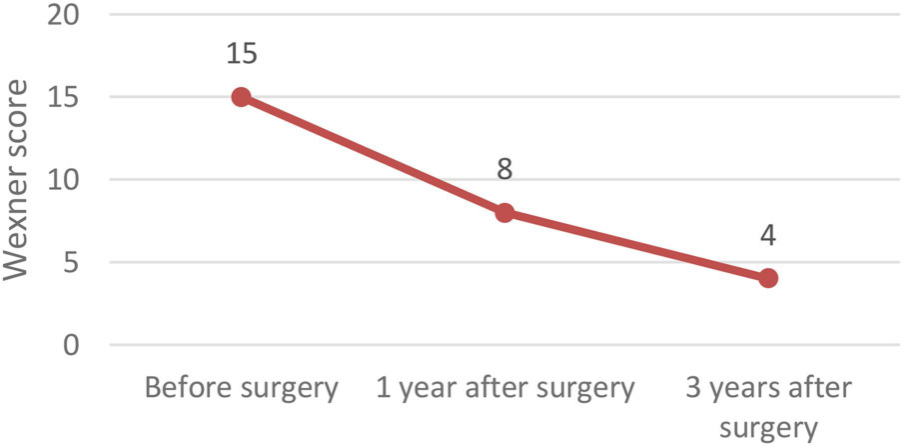
Functional outcomes – Wexner score.

**Figure 4:**
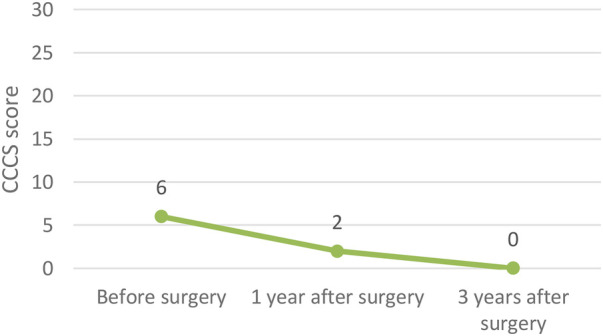
Functional outcomes – CCCS score.

In terms of functional outcomes, quality of life showed a steady improvement, with the median EQ-VAS score rising from 50 preoperatively to 68 at one year and 75 at three years postoperatively. Continence also improved, as reflected by a decrease in the median CCCS score from 15 to 8 at one year, and further to 4 at three years. Constipation symptoms declined, with the median score decreasing from 6 preoperatively to 2 at one year and reaching 0 at three years. These functional outcomes following surgery for recurrent prolapse are illustrated in Figures 2–4 and in the Table 4 (Functional outcomes—statistical analysis).

**Table 4:** Functional outcomes—statistical analysis.

Scale	Friedman *χ*^2^ (df = 2)	*p-*value	pre vs. 1 yr	pre vs. 3 yr	1 yr vs. 3 yr
EQ-VAS	12.237	0.0022	0.035	0.0086	1.000
CCCS	9.091	0.0106	0.18	0.11	0.29
Wexner	7.309	0.0259	0.017	0.029	1.000

### Complications

Surgical complications included one case of symptomatic pelvic collection and one case of anastomotic line bleeding. Both complications were treated conservatively (grade I). Among medical complications authors noted one case of upper gastrointestinal bleeding (grade III) and one case of urinary tract infection (grade II). The complications are shown in Table 5.

**Table 5:** Complications.

Surgical	
Asymptomatic pelvic collection (*n* = 1)	Clavien-Dindo grade I
Anastomotic line bleeding (*n* = 1)	Clavien-Dindo grade I
Medical	
Urinary tract infection (*n* = 1)	Clavien-Dindo grade II
Upper gastrointestinal bleeding (*n* = 1)	Clavien-Dindo grade III

## Discussion

The study indicates that the most effective methods for the treatment of recurrent rectal prolapse (RRP) are ventral mesh rectopexy (VMR) and resection rectopexy procedures. In the analyzed cohort, the success rate of VMR reached 75%. Based on these findings, the authors advocate for the use of abdominal approaches in all patients who are medically fit to undergo such procedures.

However, performing VMR in the setting of recurrence after Altemeier procedure can be technically challenging due to the shortening of the Douglas pouch, which may hinder adequate mesh coverage. Moreover, VMR for recurrent prolapse after Altemeier involves implanting the mesh on the colon, which has a thinner wall than rectum, more active peristalsis, no mesorectum, and potential diverticula—all factors that can increase the risk of mesh-related complications such as erosion, migration, or infection (6). Additionally, as with any abdominal procedure for rectal prolapse, VMR becomes more difficult following a prior VMR because of postoperative fibrosis and the possible presence of residual mesh. In the present cohort, the single recurrence following VMR consisted of a limited rectal prolapse of less than 8 cm, which was successfully managed with a Delorme procedure.

Resection rectopexy appears to be equally effective as ventral mesh rectopexy in the study, with a recurrence rate of 25%. However, it is typically reserved for cases with a redundant sigmoid colon, the presence of concomitant colonic pathology such as diverticulosis, or prolonged colonic transit time (7, 8). Interestingly, a meta-analysis conducted in 2024 did not show any statistically significant differences between VMR and resection rectopexy in terms of operation duration, length of hospital stay, complication rates, risk of surgical site infection, cardiovascular complications, resolution of constipation, or recurrence rates (9).

Certain studies indicate that the choice of surgical procedure for rectal prolapse should be guided by the type of surgery the patient has previously undergone. There is still an ongoing debate on performing a resectional procedure after a previous resectional procedure. In such cases there is a risk of ischemia of intervening segment between anastomoses. The previous anastomosis line is clearly visible during a perineal surgery, so Altemeier procedure is a safe approach after previous resection. However, performing an abdominal procedure after a previous resectional procedure can carry the aforementioned risk of ischemia, because the previous anastomosis line is not clearly visible. Thus, according to Fengler (10), performing a resection rectopexy after a previous resectional surgery is traditionally contraindicated. However, in contrary Kwang Dae Hong et al. (11) reported success in resection rectopexy following a previous resectional surgery. All in all, authors opinion is concise with the Fengler—we avoid leaving a segment of potentially devascularized proximal bowel segment, which may be prone to mucosal sloughing or in extreme cases to complete anastomotic dehiscence. However, the results published in Annals of Surgical Treatment and Research (12) show, that repeated perineal surgeries (even the non-resectional ones) can also compromise the bowel viability.

The effectiveness of secondary RP surgery appears to be lower than a primary repair in analysed cohort. Its frequency equals 25% in abdominal cases, where it was 39% in perineal cases. These results are consistent with data in the literature (3).

It is worth highlighting, that the surgery for RRP not only corrects the anatomical defect, but also drastically improves QoL. Moreover, it statistically significantly reduces fecal incontinence and constipation especially in a long-term observation.

Appropriate timing of the surgery for RRP remains unknown. In authors opinion it should not be delayed because over time the rectal prolapse leads to a worsening of fecal incontinence due to damage to the sphincter apparatus, particularly the internal anal sphincter (12). Fecal incontinence may persist even after surgery (13). Additionally, a higher recurrence rate after surgery has been documented in cases where rectal prolapse has lasted more than four years (14).

In the present cohort, no cases of mesh erosion, anastomotic dehiscence, anastomotic leak, or incomplete dehiscence were observed. One patient experienced anastomotic bleeding, which was managed conservatively. Additionally, one case of symptomatic pelvic collection (most probably seroma) occurred and was successfully treated with conservative measures.

In conclusion, the study demonstrates that both ventral mesh rectopexy (VMR) and resection rectopexy are the most effective surgical options for recurrent rectal prolapse (RRP), with comparable outcomes in terms of recurrence, complications, and postoperative recovery. Abdominal approaches should be preferred in medically fit patients due to their higher success rates and durable results. However, surgical choice must consider prior procedures, as anatomical alterations and ischemic risks may complicate resectional or repeated abdominal surgeries. While secondary repairs show lower efficacy compared to primary procedures, surgery for RRP significantly improves patients' quality of life and alleviates symptoms such as fecal incontinence and constipation. Early surgical intervention is recommended to prevent further sphincter damage and reduce the likelihood of recurrence.

## Limitations

There are numerous limitations to the conducted study. First, it is retrospective in nature. Secondly, it involves a relatively small number of patients, exclusively women. However, the analyzed condition is uncommon. Moreover, the treated patients' groups were not fully comparable—the perineal approach was utilized mostly in patients with comorbidities; thus the complication rate (especially the medical ones) can be artificially overstated. Patients were not randomly assigned to a specific surgical procedure, but it was the choice of the individual surgeon. For the VMR procedure different types of meshes (both absorbable and non-absorbable) were utilized. Finally, the follow-up of three years is relatively short, because according to Raftopoulos et al. (15) may occur up to 10 years after surgery.

## Data Availability

The original contributions presented in the study are included in the article/Supplementary Material, further inquiries can be directed to the corresponding author.

## References

[1] Pellino G Fuschillo G Simillis C Selvaggi L Signoriello G Vinci D Abdominal versus perineal approach for external rectal prolapse: systematic review with meta-analysis. BJS Open. (2022) 6(2):zrac018. 10.1093/bjsopen/zrac018 35390136 PMC8989040

[2] Fuschillo G Selvaggi L Cuellar-Gomez H Pescatori M. Comparison between perineal and abdominal approaches for the surgical treatment of recurrent external rectal prolapse: a systematic review and meta-analysis. Int J Colorectal Dis. (2025) 40(1):26. 10.1007/s00384-024-04771-z 39875708 PMC11775045

[3] Hotouras A Ribas Y Zakeri S Bhan C Wexner SD Chan CL A systematic review of the literature on the surgical management of recurrent rectal prolapse. Colorectal Dis. (2015) 17(8):657–64. 10.1111/codi.12946 25772797

[4] Daniel VT Davids JS Sturrock PR Maykel JA Phatak UR Alavi K. Getting to the bottom of treatment of rectal prolapse in the elderly: analysis of the National surgical quality improvement program (NSQIP). Am J Surg. (2019) 218(2):288–92. 10.1016/j.amjsurg.2019.02.010 30803700

[5] Bordeianou L Paquette I Johnson E Holubar SD Gaertner W Feingold DL Clinical practice guidelines for the treatment of rectal prolapse. Dis Colon Rectum. (2017) 60(11):1121–31. 10.1097/DCR.0000000000000889 28991074

[6] Schabl L Hull T Erozkan K Alipouriani A Ban KA Steele SR Ventral mesh rectopexy for recurrent rectal prolapse after Altemeier perineal proctosigmoidectomy: feasibility and outcomes. Langenbecks Arch Surg. (2024) 409(1):49. 10.1007/s00423-024-03227-w 38305915 PMC10837248

[7] Hrabe J Gurland B. Optimizing treatment for rectal prolapse. Clin Colon Rectal Surg. (2016) 29(3):271–6. 10.1055/s-0036-1584505 27582654 PMC4991961

[8] Joubert K Laryea JA. Abdominal approaches to rectal prolapse. Clin Colon Rectal Surg. (2017) 30(1):57–62. 10.1055/s-0036-1593426 28144213 PMC5179275

[9] Koimtzis G Stefanopoulos L Geropoulos G Chalklin CG Karniadakis I Alawad AA Mesh rectopexy or resection rectopexy for rectal prolapse: is there a gold standard method? A systematic review and meta-analysis. J Clin Med. (2024) 13(5):1363. 10.3390/jcm13051363 38592257 PMC10933911

[10] Fengler SA Pearl RK Prasad ML Orsay CP Cintron JR Hambrick E Management of recurrent rectal prolapse. Dis Colon Rectum. (1997) 40(7):832–4. 10.1007/BF02055442 9221862

[11] Hong KD Hyun K Um JW Yoon SG Hwang DY Shin J Clinical outcomes of surgical management for recurrent rectal prolapse: a multicenter retrospective study. Ann Surg Treat Res. (2022) 102(4):234–40. 10.4174/astr.2022.102.4.234 35475228 PMC9010966

[12] Dvorkin LS Chan CLH Knowles CH Williams NS Lunniss PJL Scott SM. Anal sphincter morphology in patients with full-thickness rectal prolapse. Dis Colon Rectum. (2004) 47(2):198–203. 10.1007/s10350-003-0035-4 15043290

[13] Cunin D Siproudhis L Desfourneaux V Berkelmans I Meunier B Bretagne J No surgery for full-thickness rectal prolapse: what happens with continence? World J Surg. (2013) 37:1297–302. 10.1007/s00268-013-1967-z 23440486

[14] Fu CW Stevenson AR. Risk factors for recurrence after laparoscopic ventral rectopexy. Dis Colon Rectum. (2017) 60:178–86. 10.1097/DCR.0000000000000710 28059914

[15] Raftopoulos Y Senagore AJ Di Giuro G Bergamaschi R. Recurrence rates after abdominal surgery for complete rectal prolapse: a multicenter pooled analysis of 643 individual patient data. Dis Colon Rectum. (2005) 48(6):1200–6. 10.1007/s10350-004-0948-6 15793635

